# ^18^F-FDG PET/CT Plays a Limited Role in Replacing Bone Marrow Biopsy for Newly Diagnosed Advanced-Stage Patients With Extranodal Natural Killer/T-Cell Lymphoma

**DOI:** 10.3389/fonc.2022.894804

**Published:** 2022-07-26

**Authors:** Chunli Yang, Wanchun Wu, Huijie Zhou, Sha Zhao, Rong Tian, Maya Xiang, Liqun Zou

**Affiliations:** ^1^ State Key Laboratory of Biotherapy and Cancer Center, West China Hospital, Sichuan University, Chengdu, China; ^2^ Department of Oncology, West China Hospital, Sichuan University, Chengdu, China; ^3^ Department of Pathology, West China Hospital, Sichuan University, Chengdu, China; ^4^ Department of Nuclear Medicine, West China Hospital, Sichuan University, Chengdu, China; ^5^ Department of Chemistry, University of Washington-Seattle Campus, Seattle, WA, United States

**Keywords:** extranodal NK/T-cell lymphoma, ^18^F-FDG PET/CT, bone marrow biopsy, bone marrow involvement, diagnosis, prognosis

## Abstract

**Purpose:**

The role of ^18^F-2-fluoro-2-deoxy-D-glucose positron emission tomography/computed tomography (^18^F-FDG PET/CT) in evaluating bone marrow (BM) involvement (BMI) among patients with extranodal natural killer/T-cell lymphoma (ENKTL) is poorly understood. This study investigated whether PET/CT could replace bone marrow biopsy (BMB) in treatment-naive ENKTL patients.

**Methods:**

Newly diagnosed ENKTL patients (n = 356) who received BMB and PET/CT to evaluate BMI at the time of diagnosis were retrospectively reviewed at West China Hospital between August 2008 and January 2020. The BMI diagnosis was confirmed using BM histology. Clinical characteristics, survival outcomes, and prognostic indicators were summarized and analyzed.

**Results:**

The cohort included 356 cases, of whom 261 were diagnosed with early-stage and 95 with advanced-stage ENKTL by PET/CT before initial treatment. No early-stage patients were identified with BMI by either BMB or PET/CT. Among the advanced-stage patients, 26 were BMB positive, and 12 of 22 patients (54.5%) with positive PET/BM results were also BMB positive. The sensitivity and specificity of PET/CT to detect BMI were 46% and 97%, respectively. The progression-free survival (PFS) and overall survival (OS) of PET/BM-negative patients were markedly longer (*p* = 0.010 and *p* = 0.001 for PFS and OS, respectively), which was consistent with the results of the BMB (*p* = 0.000 for both PFS and OS).

**Conclusion:**

Although ^18^F-FDG PET/CT showed the potential to replace BMB in the initial staging of early-stage ENKTL patients, baseline PET/CT cannot provide an accurate BMI evaluation for advanced-stage patients. A prospective study is required to confirm the diagnostic performance of BMI identification by PET/CT, along with targeted BMB and MRI for advanced-stage patients.

## Introduction

Extranodal natural kill (NK)/T-cell lymphoma (ENKTL) is an aggressive T/NK-cell neoplasm that frequently occurs among Asians and the indigenous populations of Mexico and Central and South America (1). Approximately 73–87% of patients are early-stage (stages I–II), while 13–30% are advanced-stage (stages III–IV) at the time of diagnosis (2, 3). For early-stage patients, radiotherapy combined with asparaginase-containing chemotherapy is established as the initial treatment and is associated with a 59.5–82.9% 5-year progression-free survival (PFS) rate and a 42–85.7% 5-year overall survival (OS) rate (3–7). In contrast, for patients with advanced-stage disease, asparaginase-based chemotherapy is the standard, and the 1-year PFS and OS rates are 38–86% and 57–90% (8–11), respectively. Thus, staging at diagnosis informs both the prognosis of ENKTL patients and the initial treatment strategy.

Determining the bone marrow (BM) status is critical for the initial staging of ENKTL. According to the Ann Arbor (12) and Lugano staging (13) systems, BM involvement (BMI) is associated with stage IV and prognosis is poor (14). BMI is also associated with a higher risk of hemophagocytic syndrome among patients with ENKTL (15, 16). While bone marrow biopsy (BMB) result is the gold standard used to confirm BMI, it is an invasive operation and BMB location may lead to false-negative results. Recently, ^18^F-FDG PET/CT has been widely used to assist the staging of lymphoma, including ENKTL because NK/T-cell lymphoma is intensely FDG-hypermetabolic (17). In Hodgkin lymphoma (18) and diffuse large B-cell lymphoma (19, 20), PET/CT identified BMI with high sensitivity and specificity. However, the diagnostic and prognostic performance of using PET/CT in assessing BMI among ENKTL patients remains unknown. This study aimed to investigate the concordance between PET/CT and BMB among patients with ENKTL.

## Patients and Methods

### Patients

Patients enrolled in this study were diagnosed with ENKTL using the World Health Organization classification (21). Those who were ≥ 13 years of age and were diagnosed at West China Hospital between August 2008 and January 2020 were included. Eighty-eight patients from our previous study were also included in this cohort (22). The patients had no other malignancies and received no hematopoietic growth factor injections before PET/CT or BMB. Staging procedures, including whole-body PET/CT and BMB on one side of the iliac crest, were performed for every case.

### BMB

Unilateral iliac crest marrow aspirate and trephine biopsy were routinely performed before treatment in all newly diagnosed ENKTL cases. BMB results were obtained from the clinical database and reviewed by experienced hematopathologists at diagnosis. BMB tissue was formalin-fixed, paraffin-embedded, and evaluated morphologically using a hematoxylin–eosin stain. CD3ϵ, CD20, CD56, CD5, Granzyme B staining, and Epstein–Barr virus (EBV)-encoded small RNA *in situ* hybridization (EBERs) (14) were performed in specific morphologic BMI cases.

### PET/CT Imaging and Reporting

All patients underwent whole-body ^18^F-FDG PET/CT (at least vertex or midbrain to upper thigh or foot) using a combined PET/CT scanner (Gemini GXL with a 16-slice CT component, Philips Corp., Netherlands). After a 6-hour fast and a blood glucose level of <11 mmol/L, patients were given 5.18 MBq of ^18^F-FDG per kilogram intravenously. After a 60-minute rest period, whole-body CT and PET scans were initiated. CT acquisition data were used for attenuation correction and corrected PET images were reconstructed using the line-of-response method. Syntegra software was applied for image registration and fusion.

PET/CT studies were assessed visually using PET activity in the liver blood pool as a reference. Nuclear medicine specialists carefully and separately reviewed PET/CT data to confirm BMI. The BMI assessed by PET/CT was defined as prominent focal or diffuse homogeneous ^18^F-FDG uptake in the bone marrow, exceeding that of the normal liver and without an alternative explanation relating to non-oncologic disease, trauma, or procedural history.

### Statistical Analysis and Ethics

The patients in this study were followed up by their outpatient oncologist. The diagnostic performance of PET/CT to identify BMI was summarized using the sensitivity, specificity, positive predictive value (PPV), negative predictive value (NPV), and accuracy, which were calculated using Clopper–Pearson exact confidence limits (23). The PFS and OS were summarized using the Kaplan–Meier method, and the differences were compared using the log-rank test. Univariate logistic regression analysis evaluated variables used to predict the survival of ENKTL patients. Parameters identified as statistically significant risk factors were assessed using multivariate logistic regression analysis. The PFS was calculated from the time of diagnosis to disease progression, recurrence, any cause of death, or last follow-up, and the OS was determined from the time of diagnosis to death for any reason or last follow-up. Survival data were analyzed using SPSS version 25.0 (IBM Corp., Armonk, NY, USA), and a two-sided p <0.05 was considered statistically significant. The study was performed under regulatory requirements and approved by the Ethics Committee of the West China Hospital of Sichuan University.

## Results

### Patient Characteristics

A total of 356 patients who met the study enrollment criteria were included. At diagnosis, the median age was 45 years (range, 13–77 years), and the male-to-female ratio was 1.78. Almost three-quarters (73.3%) of the cases were classified as early-stage and 26.7% were diagnosed as advanced-stage by PET/CT using the Lugano staging system (**Table 1**). Most of the patients (95.5%) presented with a nasal location as the primary lesion, and other lesions were found in the skin, stomach, and intestine. Using the Prognostic Index of Natural Killer lymphoma (PINK) score evaluation (24), 83.7% of patients were assigned to the low-risk group (**Table 1**). Using BMB detection and PET/CT scanning, 26 patients (7.3%) had ENKTL cells in the BM using BMB, whereas 22 (6.2%) tested positive by PET/CT. Patients from the BMB-positive group had a higher ratio of performance status ≥2, serum lactate dehydrogenase (LDH), and B symptoms than BMB-negative patients (**Table 2**).

**Table 1:** Characteristics of ENKTL patients at diagnosis in this study (n = 356).

Characteristics	No. of patients	%
**Male:female ratio**	1.78:1	
**Age, years**		
Median	45	
Range	13-77	
**Lugano stage**		
I-II	261	73.3
III-IV	95	26.7
**ECOG PS**		
0-1	274	77.0
≥2	82	33.0
**B symptoms**		
Yes	203	57.0
No	153	43.0
**Elevated serum LDH**		
Yes	147	57.4
No	209	42.6
**Plasma EBV DNA copies**		
Positive	206	57.9
Negative	84	23.6
Unknown	66	18.5
**PINK score**		
0	218	61.2
1	80	22.5
2	48	13.5
3	9	2.5
4	1	0.3
**Primary site**		
Nose	340	95.5
Skin	7	2.0
Gastric and intestine	6	1.7
Other	3	0.8
**BMB positive**		
Yes	26	7.3
No	330	92.7
**PET/BM positive**		
Yes	22	6.2
No	336	93.8

ECOG, Eastern Cooperative Oncology Group; PS, performance status; EBV, Epstein-Barr virus; LDH, lactate dehydrogenase; PINK, Prognostic Index of Natural Killer lymphoma; BMB, bone marrow biopsy; PET, positron emission tomography; BM, bone marrow.

N=356.

**Table 2:** Baseline features of patients with bone marrow involvement versus. patients without bone marrow involvement by BMB (n = 356).

Charateristics	No. of patients with BMB positive, N = 26, %	No. of patients with BMB negative, N = 330, %
**Male: female ratio**	2.25:1	1.75:1
**Age, years**		
Median	45	45
Range	23-66	13-77
**ECOG PS**		
0-1	8(30.8)	266 (80.6)
≥2	18(64.3)	64 (19.4)
**B symptoms**		
Yes	23(88.5)	180 (54.5)
No	3(11.5)	150 (45.5)
**Serum LDH increase**		
Yes	24(92.3)	123 (37.3)
No	2(7.7)	207 (62.7)
**Plasma EBV DNA copies**		
Positive	21(80.8)	185 (56.1)
Negative	0	85 (25.8)
Unknown	5(19.2)	60 (18.2)
**PINK score**		
0	2(7.7)	216 (65.5)
1	14(53.8)	66 (20.0)
2	10(38.5)	38 (11.5)
3	0	9 (2.7)
4	0	1 (0.3)
**Primary site**		
Nasal	22(84.6)	318 (96.4)
Skin	1(3.8)	5 (1.5)
Gastrointestinal	2(7.7)	5 (1.5)
Other	1(3.8)	2 (0.6)
**PET/BM positive**		
Yes	12(46.2)	10 (3.1)
No	14(53.8)	310 (96.9)

ECOG, Eastern Cooperative Oncology Group; PS, performance status; LDH, lactate dehydrogenase; PINK, Prognostic Index of Natural Killer lymphoma; BMB, bone marrow biopsy; PET, positron emission tomography; BM, bone marrow.

N=356.

### Diagnostic Performance of PET/CT

**Figure 1:**
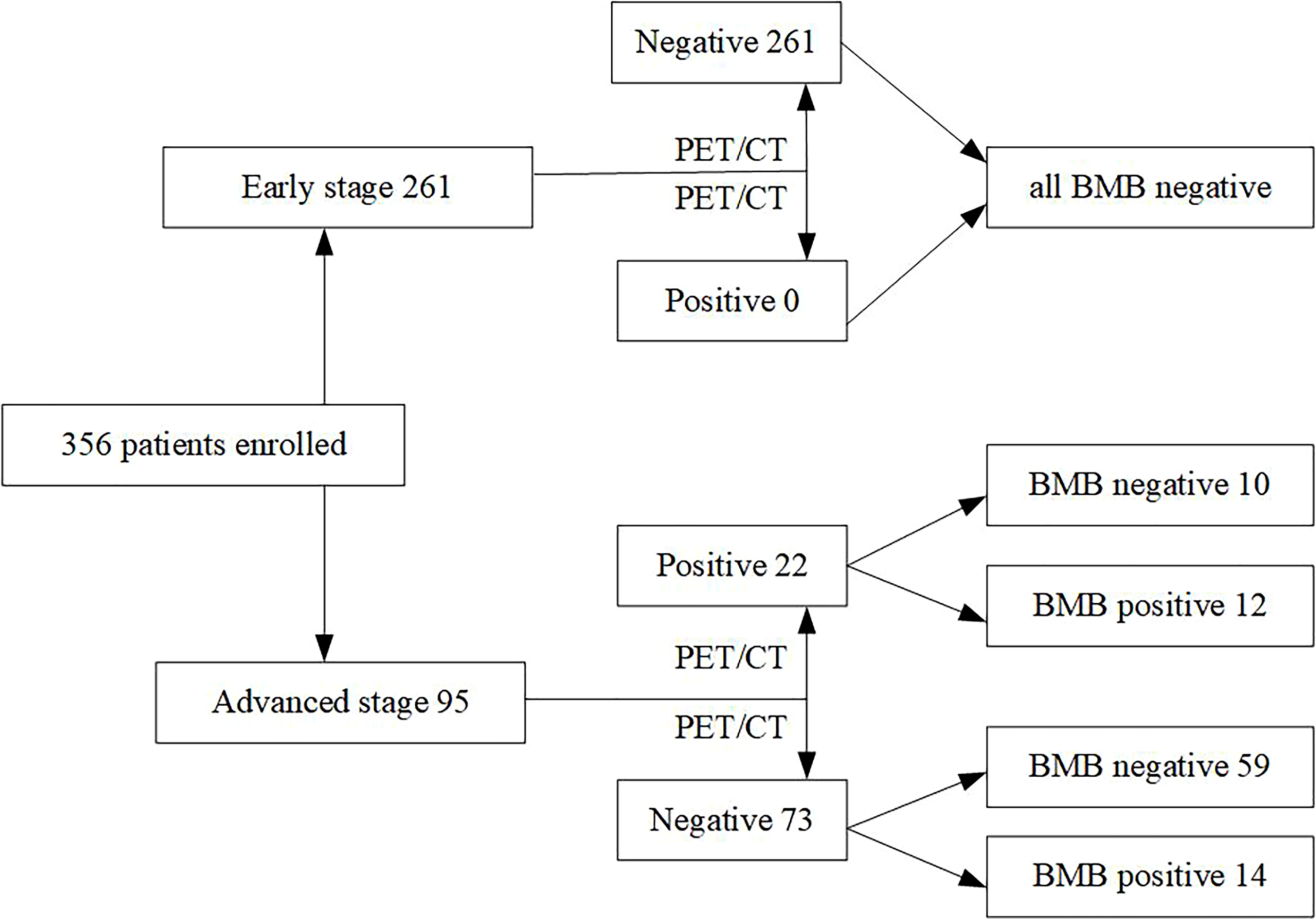
The distribution of bone marrow status as assessed with PET/CT and bone marrow biopsy ENKTL patients.

PET/CT and BMB were used to detect the BM status at the time of diagnosis for 261 early-stage and 95 advanced-stage patients (**Figure 1**). Among early-stage patients, PET/CT detected that 261/261 (100%) were BM negative. Among advanced-stage patients, 22/95 (23.2%) were PET/BM-positive, and 73/95 (76.8%) were negative. Of the PET/BM-positive cases, 12/22 (54.5%) were BMB positive. Additionally, 14/73 (19.2%) of advanced-stage patients with PET/BM negative were BMB positive. PET/CT detected BMI in 12 of the 26 BMB-positive patients (46.2% sensitivity; **Table 3**). Of the 330 patients who were PET/BM negative, 320 were also negative using BMB (96.9% specificity; **Table 3**). The positive predictive value (PPV) and negative predictive value (NPV) of PET/CT for identifying BMI were 54.5% and 95.8%, respectively, and the positive and negative likelihood ratios were 15.2 and 0.6, respectively. The BM examination by PET/CT did not change the clinical stage between early- and advanced-stage and the initial treatment strategy of the cohort.

**Table 3:** Comparison of the diagnostic performance of PET/CT in bone marrow involvement.

Diagnostic Modality	BMB postive, N=26	
	Value	95% CI
**PET/CT**		
Sensitivity	46.2%	27.1%-66.3%
Specificity	96.9%	94.3%-98.5%
PPV	54.5%	32.7%-74.9%
NPV	95.8%	92.9%-97.6%
Accuracy	93.2%	90.2%-95.4%
Positive likelihood ratio	15.2	7.3-31.9
Negative likelihood ratio	0.6	0.4-0.8

BMB, bone marrow biopsy; PET, positron emission tomography; CT, computed tomography; PPV, positive predictive value; NPV, negative predictive value.

### Prognostic Performance of PET/CT

**Figure 2:**
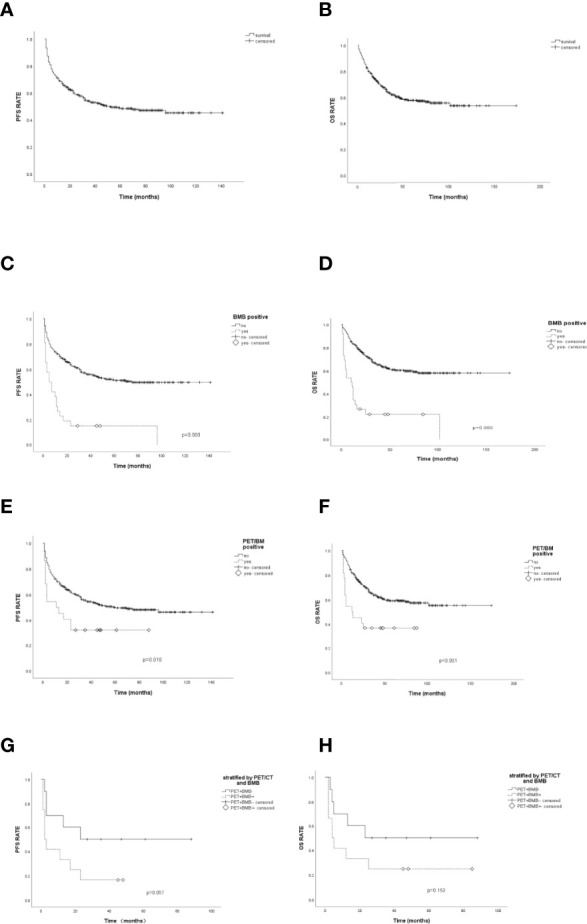
Progression-free survival (PFS) and overall survival (OS) analysis. **(A, B)**: The median PFS (mPFS) and median OS (mOS) of 356 patients was 51.0 months and 144.0 months. **(C)** The mPFS of bone marrow biopsy (BMB) positive was 5.0 months, and 75.0 months for negative patients, *p*=0.000. **(D)**. The mOS of BMB positive was 10.0 months, and 144.0 months for negative patients, *p*=0.000. **(E)** The mPFS of PET/BM positive was 11.0 months, and 60.0 months for negative patients, *p*=0.010. **(F)** The mOS of PET/BM positive was 12.0 months, and 144 months for negative patients, *p*=0.001. **(G)** The mPFS of patients with PET/BM false positive (PET+BMB-) and PET/BM true positive (PET+BMB+) were 23.0 months and 2.0 months, respectively, *p*=0.057. **(H)** The mOS of patients with PET/BM false and true positive were 23.0 months and 4.0 months, respectively, *p*=0.163.

The median follow-up time for this cohort was 59.0 months (range, 50.1–67.1 months). During this time, the median PFS (mPFS) was 51.0 months (95% CI, 30.0–72.0 months, **Figure 2A**), and the median OS (mOS) was 144.0 months (95% CI, 61.3–226.7 months, **Figure 2B**). The BMB-positive group had mPFS of 5.0 months (95% CI, 0.0–10.0 months; *p* = 0.000) and mOS of 10.0 months (95% CI, 4.3–15.7 months; *p* = 0.000) (**Figures 2C, D** **)**. Survival differed significantly between the PET/BM-positive and negative groups (**Figures 2E, F** **)**. The mPFS was 60.0 months (95% CI, 37.3–82.7 months) for the PET/BM-negative group and only 11.0 months (95% CI, 0.0–22.5 months) for the positive group (*p* = 0.010). Similarly, the mOS was 144.0 months (95% CI, 61.3–226.7 months) for the PET/BM-negative group and only 12.0 months (95% CI, 0.0–29.5 months) for the PET/BM-positive group (*p* = 0.001).

Survival outcomes following combined PET/CT and BMB were also investigated. For the PFS evaluation (**Figure 2G**), the ten PET/BM false positive patients (PET/BM positive but BMB negative, PET+BMB−) had an mPFS of 23.0 months (95% CI not estimated), while PET/BM true positive (both PET/BM-positive and BMB-positive, PET+BMB+) patients had an mPFS of 2.0 months (95% CI, 0.3–3.7 months) (*p* = 0.057). Similarly, the mOS of PET/BM false positive cases was 23.0 months (95% CI not estimated), while the mOS of PET/BM true positive patients was 4.0 months (95% CI, 0.6–7.4 months) (*p* = 0.163) (**Figure 2H**).

Univariate and multivariate analyses were also performed for this cohort. In the univariate analyses, the Eastern Cooperative Oncology Group (ECOG) performance status (PS) score ≥2, elevated serum LDH, the Lugano stages III–IV, the PINK score ≥2, and BMI using BMB or PET/CT were significantly associated with inferior PFS and OS (**Table 4**). The parameters identified as statistically significant risk factors (*p <*0.05) were assessed by multivariate logistic regression and collinearity analysis was performed between the variables in the Cox proportional-hazards model. Patients with an ECOG PS score of ≥2 (RR 1.494, *p* = 0.046), elevated serum LDH (RR 1.958, *p* = 0.011), and a positive BMB (RR 1.768, *p* = 0.044) had inferior PFS, while those with elevated serum LDH (RR 1.686, *p* = 0.007) and a positive BMB (RR 2.060, *p* = 0.019) had shorter OS (**Table 5**).

**Table 4:** Univariate analysis of PFS and OS in 356 patients with ENKTL.

Variable	PFS			OS		
	HR	*95% CI*	*P*	HR	*95% CI*	*P*
**Age** **(>60 years)**	0.794	0.508-1.242	0.312	0.772	0.465-1.281	0.316
**ECOG PS (≥2)**	2.545	1.844-3.512	**0.000**	2.749	1.932-3.914	**0.000**
**Elevated serum LDH**	2.165	1.610-2.913	**0.000**	2.473	1.777-3.443	**0.000**
**Lugano Stage (III-IV)**	2.667	1.969-3.612	**0.000**	2.678	1.921-3.737	**0.000**
**PINK score (≥2)**	1.597	1.332-1.915	**0.000**	1.619	1.330-1.970	**0.000**
**BMI by BMB**	3.360	2.156-5.239	**0.000**	4.285	2.682-6.846	**0.000**
**BMI by PET/CT**	1.958	1.152-3.327	**0.013**	2.493	1.434-4.334	**0.001**

ECOG, Eastern Cooperative Oncology Group; PS, performance status; LDH, lactate dehydrogenase; PINK, Prognostic Index of Natural Killer lymphoma; BMI, bone marrow involvement; BMB, bone marrow biopsy; PET, positron emission tomography; CT, computed tomography. The meaning of bold values/numbers used a is the p-value less than 0.05.

**Table 5:** Multivariate analysis of PFS and OS in 356 patients with ENKTL.

	PFS		OS	
Variable	RR (95% CI)	*P*	RR (95% CI)	*P*
**ECOG PS ≥2**	1.494 (1.007-2.216)	**0.046**	1.374 (0.894-2.111)	0.148
**Elevated serum LDH**	1.559 (1.108-2.193)	**0.011**	1.686 (1.157-2.458)	**0.007**
**Lugano stage (III-IV)**	1.383 (0.714-2.678)	0.336	1.374 (0.894-2.111)	0.078
**PINK score (≥2)**	1.559 (0.858-2.272)	0.437	0.974 (0.652-1.455)	0.898
**BMI by BMB**	1.768 (1.015-3.081)	**0.044**	2.060 (1.127-3.766)	**0.019**
**BMI by PET/CT**	0.765 (0.413-1.418)	0.395	0.874 (0.450-1.701)	0.693

ECOG, Eastern Cooperative Oncology Group; PS, performance status; LDH, lactate dehydrogenase; PINK, Prognostic Index of Natural Killer lymphoma; BMI, bone marrow involvement; BMB, bone marrow biopsy; PET, positron emission tomography; CT, computed tomography. The meaning of bold values/numbers used is the p-value less than 0.05.

## Discussion

This study evaluated the use of PET/CT to detect BMI and assessed whether it could replace BMB for the initial staging of ENKTL. A positive BMB result was applied as the standard criterion for BMI. The findings showed that while the specificity was 96.9%, the sensitivity was only 46.2%. Like the prognosis performance of BMB, PET/BM-negative patients had better PFS and OS than PET/BM-positive patients. To our knowledge, this is the largest series to date that has examined the ability of PET/CT to detect BMI in ENKTL patients undergoing BMB staging.

By BMB, ENKTL infiltrates the BM in up to 7% of patients (25, 26). In the study cohort, 7.3% of all patients and 27.4% of advanced-stage patients had BMI according to BMB. Because of its high sensitivity, PET/CT has become a frequently used tool for staging, response evaluation, and follow-up after treatment of patients with ENKTL. Suchitra et al. (27) reported that among 60 T/NK-cell lymphoma patients, which only included seven cases of ENKTL, PET/CT had 53.3% sensitivity and 100% specificity to detect BMI. Three published studies have evaluated the use of PET/CT to detect BMI among patients with ENKTL. One study (28) of 55 ENKTL patients showed that 5/12 PET/BM-positive patients were confirmed by BMB and the true positivity and negativity were 100 and 86%, respectively. Another study (22) found that four patients with advanced-stage ENKTL with BMI by PET/CT were BMB positive, indicating a sensitivity and specificity of 100 and 92.8%, respectively. Youngil et al. reported (29) that among 109 patients with T/NK-cell lymphoma, including 46 ENKTL, the sensitivity and specificity of PET/CT for diagnosing BMI in ENKTL were 58.3% and 85.3% by visual analysis.

In this study, the sensitivity of 46.2% was much lower than that reported previously, while the specificity of 96.9% was higher (22, 27, 28). This may be because the sample size used in this study was much larger than that used in previous studies (22, 27, 28). Additionally, the current study diagnosed 26 patients with BMI by BMB and 22 by PET/CT, which was more than five times as many as were identified previously (22). The only true positive results ever reported were also BM positive and contained bone lesions using both PET/CT and BMB (22). Of the 22 patients who were PET/BM positive in this study, the lesion sites included the sternum, ribs, humerus, pelvis, femur, and tibia. However, the BMB was performed only by unilateral iliac crest biopsy, which may cause false-negative results. Future studies should emphasize that BMI locations other than the iliac crest identified by PET/CT should be confirmed by targeted biopsy or complementary magnetic resonance imaging (MRI) in advanced-stage patients.

BMI identified by BMB is an indicator of poor prognosis among ENKTL patients (14, 22, 28). The current study supports prior findings that BMB-positive patients have inferior PFS and OS. However, few studies have assessed the prognosis of PET/BM-positive patients on a large scale. This study found that PET/BM-negative patients also had longer mPFS and mOS than PET/BM-positive patients, supporting the results of smaller sample-sized studies (22, 28). Prognostic performance was also assessed for combined PET/BM and BMB. Although the PET/BM false positives tended to have a better prognosis, the differences were not significant. This may be because, although being the largest to date, the sample size was too small to detect a difference. It is also possible that there were more than 12 PET/BM true positive patients because the targeted BMB and the MRI results were not considered, which may have affected the results. Consistent with previous results (22), BMI detected by PET/CT was associated with inferior survival in the univariate analysis. The significant prognostic difference between PET/BM-positive and negative patients disappeared in the COX proportional hazard model (*p* = 0.395 for mPFS and *p* = 0.693 for mOS) but remained for BMB-positive and negative patients, indicating that BMB has a better prognostic performance for BMI than PET/CT.

Based on these findings, we suggest that when PET/CT staging is limited to stages I–II, the BM evaluation by BMB can be omitted on the basis of the strong specificity of PET/CT. Otherwise, the BM evaluation by BMB should be used to exclude false results regardless of the PET/BM status. A future prospective randomized trial should verify these results.

This study had several limitations. First, the BMB was performed only at the iliac crest and for BMI suspected in other locations by PET/CT, like the sternum and femur, targeted BMB or MRI were not conducted, potentially decreasing PET/BM sensitivity. Second, this study was retrospective, so the data are likely to include some selection and information bias. Third, the data included patients receiving a range of first-line treatments that may have an unexpected bias on survival outcomes.

In conclusion, ^18^F-FDG PET/CT showed the potential to replace BMB for the initial staging of early-stage ENKTL patients, while baseline PET/CT could not provide an accurate BMI evaluation for advanced-stage patients. A prospective study is needed to confirm the diagnostic performance of BMI identification by PET/CT combined with targeted BMB and MRI for advanced-stage patients.

## Data Availability Statement

The raw data supporting the conclusions of this article will be made available by the authors, without undue reservation.

## Ethics Statement

The studies involving human participants were reviewed and approved by the Ethics Committee of West China Hospital of Sichuan University. Written informed consent to participate in this study was provided by the participants’ legal guardian/next of kin.

## Author Contributions

LZ: Conception and design. CY, WW, HZ, and MY: Collection and analysis of data. SZ: Pathological review. RT: PET/CT review. All authors listed have made a substantial, direct, and intellectual contribution to the work and approved it for publication.

## Conflict of Interest

The authors declare that the research was conducted in the absence of any commercial or financial relationships that could be construed as a potential conflict of interest.

## Publisher’s Note

All claims expressed in this article are solely those of the authors and do not necessarily represent those of their affiliated organizations, or those of the publisher, the editors and the reviewers. Any product that may be evaluated in this article, or claim that may be made by its manufacturer, is not guaranteed or endorsed by the publisher.
